# Extraction of chemical-induced diseases using prior knowledge and textual information

**DOI:** 10.1093/database/baw046

**Published:** 2016-04-14

**Authors:** Ewoud Pons, Benedikt F.H. Becker, Saber A. Akhondi, Zubair Afzal, Erik M. van Mulligen, Jan A. Kors

**Affiliations:** 1 Department of Medical Informatics; 2 Department of Radiology, Erasmus University Medical Center, 3000 DR Rotterdam, PO Box 2040, The Netherlands

## Abstract

We describe our approach to the chemical–disease relation (CDR) task in the BioCreative V challenge. The CDR task consists of two subtasks: automatic disease-named entity recognition and normalization (DNER), and extraction of chemical-induced diseases (CIDs) from Medline abstracts. For the DNER subtask, we used our concept recognition tool Peregrine, in combination with several optimization steps. For the CID subtask, our system, which we named RELigator, was trained on a rich feature set, comprising features derived from a graph database containing prior knowledge about chemicals and diseases, and linguistic and statistical features derived from the abstracts in the CDR training corpus. We describe the systems that were developed and present evaluation results for both subtasks on the CDR test set. For DNER, our Peregrine system reached an *F*-score of 0.757. For CID, the system achieved an *F*-score of 0.526, which ranked second among 18 participating teams. Several post-challenge modifications of the systems resulted in substantially improved *F*-scores (0.828 for DNER and 0.602 for CID). RELigator is available as a web service at http://biosemantics.org/index.php/software/religator.

## Introduction

The extraction of chemicals, diseases, and their relationships from unstructured scientific publications is important for many areas of biomedical research, such as pharmacovigilance and drug repositioning (1, 2). Text-mining systems in combination with methods for literature-based discovery and network analysis hold promise for automatically generating new hypotheses and fresh insights (3, 4). The manual extraction of these entities and relations, and their storage in structured databases is cumbersome and expensive, and it is impossible for researchers or curators to keep pace with the ever-swelling number of papers that are being published. Automatic extraction of chemical–disease relations (CDRs) should solve these problems, but previous attempts have met with limited success. One of the difficulties that has to be addressed is the identification of relevant concepts, i.e. chemicals and diseases (5, 6). Concept identification goes beyond concept recognition in that not only the mention of a chemical or a disease has to be recognized, but that in addition a unique identifier has to be assigned, which links the concept to a source that contains further information about it (7). Also the detection of relationships between the identified chemicals and diseases remains a challenging task (8–10), partly because available annotated corpora to train and evaluate extraction algorithms are limited in size (11, 12).

In BioCreative V, one of the challenge tasks is the automatic extraction of CDRs from biomedical literature (13). The CDR task comprises two subtasks. The first subtask involves automatic disease-named entity recognition and normalization (DNER) from a set of Medline documents, and can be considered as a first step in CDR extraction. The second subtask consists of extracting chemical-induced diseases (CIDs) and delivering the chemical-disease pairs per document.

Our team participated in both CDR subtasks. For the DNER subtask, we used our concept recognition tool Peregrine (14), in combination with several optimization steps. For the CID subtask, we applied the optimized Peregrine system for disease concept recognition; for chemical concept recognition, we used tmChem (15), a chemical concept recognizer that was provided by the challenge organizers. A relation extraction module was trained on a rich feature set, including features derived from a graph database containing prior knowledge about chemicals and diseases, and linguistic and statistical features derived from the training corpus documents.

In the following, we describe the systems that we developed for the BioCreative challenge, as well as several post-challenge improvements, and present evaluation results for both subtasks on the CDR training and test sets.

## Methods

**Figure 1:**
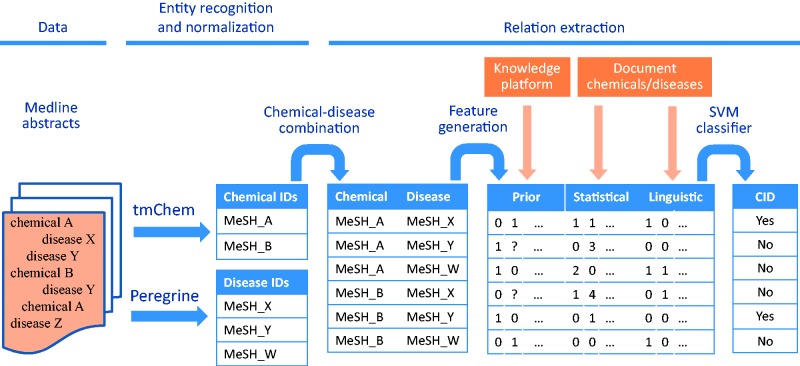
Workflow for CDR extraction. The chemical and disease entities in a Medline abstract are recognized and mapped to their corresponding MeSH identifiers by tmChem (for chemicals) and Peregrine (for diseases). For each possible combination of chemicals and diseases that are found in the document, features are generated based on prior knowledge from a knowledge platform, and based on statistical and linguistic information from the document. The features are fed to an SVM classifier to detect CIDs.

Figure 1 shows the different steps in our workflow for CDR extraction from biomedical abstracts. The data, methods for entity recognition and normalization, and relation extraction methods are described below.

### Data

The CDR task data consist of a training, a development and a test set, each containing 500 Medline abstracts. Chemicals and diseases in the abstracts were manually annotated in the form of text offset, text span, and Medical Subject Headings (MeSHs) identifier (13). Chemical-disease interactions were annotated at the document level as MeSH-identifier pairs, but only if a mechanistic relationship between a chemical and a disease was explicitly mentioned in the abstract (16). Therapeutic relationships between chemicals and diseases were not annotated. Table 1 shows the number of annotated (unique) identifiers of chemicals and diseases, and the number of annotated relationships.

**Table 1:** Characteristics of the CDR corpus

Data	Training	Development	Test	Total
Abstracts	500	500	500	1500
Chemical mentions	5203	5347	5385	15 935
Unique chemical identifiers	1467	1507	1435	4409
Disease mentions	4182	4244	4424	12 850
Unique disease identifiers	1965	1865	1988	5718
CDRs	1038	1012	1066	3116

### Entity Recognition and Normalization

Chemical concept recognition was carried out using the tmChem chemical recognizer system (15). The tmChem system was one of the best performing systems in the previous BioCreative IV chemical-named entity recognition (CHEMDNER) challenge (17). It includes a dictionary look-up to map recognized chemicals to MeSH identifiers. tmChem is an ensemble system that combines two CRF-based systems, of which we only used the one that performed best in the CHEMDNER challenge. We trained this system on the 1000 documents in the CDR training and development sets.

For the recognition and normalization of diseases, we employed our dictionary-based concept recognition system Peregrine (14). Peregrine employs a user-supplied dictionary and splits the terms in the dictionary into sequences of tokens. When such a sequence of tokens is found in a document, the term and the concept associated with that term, is recognized in the document. Peregrine removes stopwords (we used the PubMed stopword list [http://www.ncbi.nlm.nih.gov/books/NBK3827/table/pubmedhelp.T.stopwords]) and tries to match the longest possible text phrase to a concept. It uses the Lexical Variant Generator tool of the National Library of Medicine to reduce a token to its stem before matching (18). Peregrine is freely available (https://trac.nbic.nl/data-mining/).

We constructed a dictionary with concepts and corresponding terms taken from four biomedical vocabularies, as contained in the Unified Medical Language System (UMLS) (19) 2015AA edition. These are: MeSH; Medical Dictionary for Regulatory Activities; Systematized Nomenclature of Medicine, Clinical Terms, and International Classification of Diseases, Tenth Edition, Clinical Modification. The MetamorphoSys tool (19) was used to only include concepts that belong to the semantic group ‘Disorders’ (20), and to discard terms that are flagged as suppressible in the UMLS.

After a document was processed with Peregrine, several post-processing steps were executed. We extracted all abbreviations and their corresponding long forms (21), and made sure that any combination of abbreviation and long form was tagged with the same concept. Adjacent term spans that were identified as the same concept were merged. For our challenge submission on the test set, we filtered out terms that Peregrine had tagged erroneously in the training and development data (false-positive terms).

After the challenge, we refined this approach by only removing false-positive terms if the ratio of true-positive to false-positive terms was lower than 0.3. This threshold was heuristically set based on the training data to prevent that an occasional false-positive detection would cause the removal of terms that were generally correctly recognized. Moreover, we performed a term-frequency analysis by indexing a random set of one million Medline abstracts and manually checking the 2000 top-ranking terms found by Peregrine. Erroneously recognized terms were also removed. Finally, we added all terms that Peregrine had missed in the training set (false-negative terms) to the dictionary.

The UMLS identifiers of the concepts that resulted from the indexing and post-processing steps were mapped to MeSH identifiers with the IntraMap tool (22). IntraMap contains a precompiled mapping table that links each UMLS concept to the semantically closest MeSH header.

### Relation Extraction

We formulated the relation extraction task as a binary decision problem: for each possible pair of chemicals and diseases found in a document, determine whether there is a relationship. To train the relation extraction algorithm, we constructed training instances based on the perfect (gold-standard) entity annotations of the training data. Of the 10 693 possible pairs of annotated chemicals and diseases, 2050 were labeled as positive instances because the pair had been annotated as a relationship by the reference. The other 8643 pairs were labeled as negative instances. Co-occurrence pairs were allowed to cross the title-abstract border. For each instance, three sets of features were generated, based on prior knowledge and on statistical and linguistic information from the document.

### Prior knowledge features

To generate features based on existing, prior knowledge, we used a graph database, the Euretos Knowledge Platform (http://euretos.com/). The Euretos Knowledge Platform is a commercial system and not freely available, but life-science researchers can request free browsing access. We have obtained an academic license to use a local installation of the system. The graph database contains entities and relations from (curated) structured databases, such as UniProt, the Comparative Toxicogenomics Database and UMLS, and from scientific abstracts (semantic Medline (23)). Each connection between entities can have a set of named relations or predicates. Attached to each predicate is provenance information, including the different sources in which the relation was found and, per source, the number of records or abstracts with the relation. Euretos provides an application programming interface that was used to query the database for paths between two given entities. A path can be direct (i.e. the entities have a direct, one-directional (causal) or two-directional (non-causal), relationship) or indirect (the entities are connected through one intermediate entity; if the two relationships involved are one-directional, one relationship should point towards the intermediate entity and the other should point away from it). For each path, a confidence score based on provenance information is computed that indicates how strongly the entities are related. If two entities are connected through both direct and indirect paths, the latter are ignored. If there are multiple paths of the same length, the total score and total provenance count are taken as the maximum of the path scores and path provenance counts, respectively. The provenance count of an indirect path is taken as the minimum of the provenance counts of the two predicates involved. For each chemical-disease pair, we determined the path type (direct, indirect or no path), the confidence score, the number of paths, the set of predicates involved and the provenance count.

### Statistical features

The statistical feature set contains, for each chemical-disease pair at the document level, the number of mentions of the chemical and of the disease and number of possible chemical-disease pairs in the document (i.e. number of chemical mentions times number of disease mentions). The ratios of these numbers to the numbers of all chemical mentions, all disease mentions and all possible chemical-disease pairs in the document are also taken as features. Additional features capture the minimal sentence and word distance between the mentions of the chemical and the disease. Binary features indicate whether the chemical, the disease or both were mentioned in the document title. The MeSH identifiers of the chemical and disease are included as nominal features.

### Linguistic features

**Figure 2:**
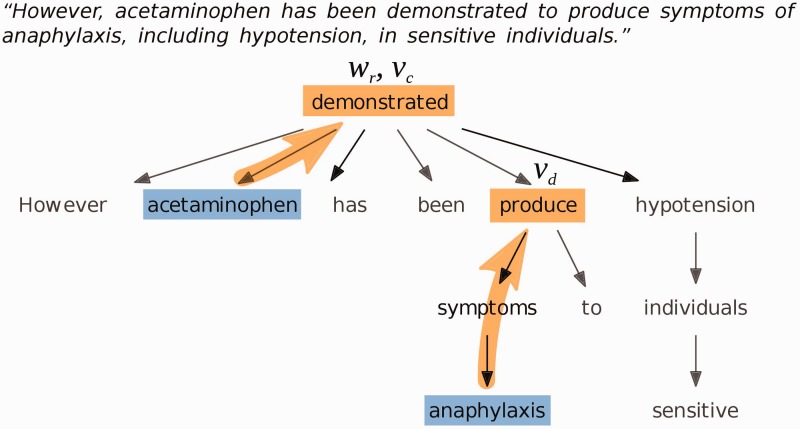
Example dependency parse tree for a sentence about the chemical ‘acetaminophen’ and the disease ‘anaphylaxis’. The governing verb of the disease is ‘produce’; the governing verb of the chemical is ‘demonstrated’, which is also the relating word.

We used the Stanford CoreNLP parser version 3.4.1 with the English PCFG parsing module to generate dependency trees of the sentences of each document, and determined ‘governing verbs’ of chemicals and diseases, and ‘relating words’ of chemical-disease pairs (Figure 2). The governing verb of a word is defined as the first verb in the parse tree that is encountered when the tree is traversed from the word towards the root. The relating word of a chemical-disease pair is defined as the first word in the parse tree that the chemical and disease have in common. If the chemical and disease mentions appear in different sentences, the relating word is undefined.

Two sets of linguistic features are used. For the first set, only one pair of chemical and disease mentions in the document is considered. The pair is selected on the basis of the following heuristics. A pair with the chemical and disease mentions in the same sentence has precedence over a pair with mentions in different sentences, and a pair where no other chemical-disease pair can be found lower in the parse tree has precedence over a pair for which this is not true. If there are only pairs with mentions in different sentences, the last pair with the chemical mention before the disease mention is selected. If no such pair exists, the first chemical and disease mentions in the document are selected. The following features are derived: governing verb of the chemical and of the disease, relating word, and governing verb of the relating word. Note that if the chemical and the disease occur in different sentences, the governing verbs are taken from different parse trees and the relating word and its governing verb are undefined. Further features indicate whether the chemical is mentioned before the disease, and whether another chemical-disease pair can be found lower in the parse tree. After the challenge, we added four features that signify whether the relating word and the governing verb of the chemical, of the disease, and of the relating word, are negated. Negation was assessed by the presence of negation modifiers in the parse tree. Three more post-challenge features indicate whether the chemical is the same as the relating word, whether the governing verb of the disease is the same as the relating word, and whether both governing verbs are the same as the relating word.

For the second set of linguistic features, we aggregated information about the governing verbs and relating words from all possible pairs of chemical and disease mentions in the documents. This set contains one numeric feature for each governing verb or relating word encountered in the training set, indicating how many times that word is found as a governing verb or relation word for the chemical-disease pairs in the document.

### Machine learning

Various machine-learning algorithms were explored, utilizing Weka machine learning libraries (http://www.cs.waikato.ac.nz/ml/weka/). Performance was estimated by 10-fold cross-validation.

In a preliminary analysis in which we compared various classification algorithms, support vector machines (SVMs) proved to have superior performance. Therefore, we continued to optimize parameters for the SVM classification model. We used C-support vector classification with radial basis function kernel type, initially with default settings for cost (1.0) and gamma (0.0).

All numeric features were normalized to scale between zero and one. Because of the class imbalance the cost matrix of the SVM was set to 5:1, giving extra weight to the minority class. Utilizing the best performing feature set, we tuned the cost and gamma parameters by performing a grid search, again applying 10-fold cross-validation. During the grid search, we used a fixed decision threshold of 0.5 for the SVM. We subsequently varied the decision threshold to optimize the *F*-score of the SVM.

### Evaluation

For each document, the disease concepts and the disease–chemical relationships found by our systems were compared with the gold-standard annotations, resulting in true-positive, false-positive and false-negative detections. Micro-averaged precision, recall and *F*-score were then computed over the whole document set. We implemented our final challenge systems as web services, which the CDR task organizers utilized for online system evaluation on the test set.

## Results

### DNER task

Table 2 shows the performance of the Peregrine challenge system and the system with post-challenge modifications on the DNER test set. The challenge system obtained an *F*-score of 0.757. The modified system performed considerably better achieving an F-score of 0.828, well above the average *F*-score (0.760) of the 16 teams participating in the DNER task (13).

**Table 2:** Performance of the Peregrine challenge and post-challenge systems for disease normalization on the test set

System	Recall	Precision	F-score
Peregrine, challenge	0.772	0.737	0.757
Peregrine, post challenge	0.839	0.818	0.828

To get insight in the cause of the remaining errors of the modified Peregrine system, we randomly selected and analyzed 50 false-positive and 50 false-negative detections. Table 3 shows that almost half of the false-positives were due to incorrectly recognized terms, e.g. in the form of an erroneous synonym (‘patch’ for ‘plaque’) or a term that is no disease (‘glucose tolerance curve’). The largest group of false-negatives resulted from missing synonyms in the terminology. Interestingly, many of these synonyms were present in other vocabularies in the UMLS than the ones that we selected for building our terminology. Smaller number terms were correctly recognized, but were mapped to the wrong MeSH identifier, or were excluded because their true-/false-positive ratio was below the threshold of 0.3. Partial recognition of terms, e.g. ‘carcinoma’ in ‘cervical carcinoma’ or ‘ethanol abuse’ in ‘cocaine and ethanol abuse’, resulted in considerable numbers of false-positives as well as false-negatives. Finally, we encountered a number of gold-standard annotation errors. For example, in the term ‘ST depression’, an electrocardiographic concept, ‘depression’ had been annotated as a psychological disorder. As another example, a mention of the term ‘death’ had not been annotated, whereas the annotation guidelines explicitly state that this term should be annotated.

**Table 3:** Error analysis of 50 false-positive and 50 false-negative errors of the post-challenge Peregrine system

Error type	False-positive	False-negative
Term mapped to incorrect MeSH identifier	8	6
Term incorrectly on exclusion list	-	5
Term partially recognized	13	15
Term incorrectly recognized	23	-
Term not recognized	-	20
Annotation error	6	4

### CID Task

Table 4 shows the results of different relation extraction systems on the CDR training and development data, using the gold-standard chemical and disease annotations to generate all possible chemical-disease pairs.

**Table 4:** Performance of different relation extraction systems on the CDR training and development data, given perfect entity annotations

System	Threshold*	Recall	Precision	F-score
Co-occurrence at sentence level	n/a	0.725	0.313	0.437
Knowledge base	n/a	0.664	0.405	0.503
SVM, all challenge features	0.30	0.840	0.693	0.760
SVM, all post-challenge features	0.34	0.854	0.753	0.801
without prior knowledge features	0.33	0.765	0.695	0.728
without statistical features	0.39	0.775	0.683	0.726
without linguistic features	0.38	0.842	0.701	0.765

* Probability threshold for the SVM to decide whether there is a relationship.

A baseline system based on sentence co-occurrence of entities gave an *F*-score of 0.437 with a recall of 0.725, indicating that more than a quarter of the relations spanned more than one sentence. The application of prior knowledge, assuming that a relation was present if a chemical and a disease were directly connected in the Euretos Knowledge Platform by a non-treatment predicate, resulted in an *F*-score of 0.503. When the SVM was trained with all the challenge features (i.e. without the negation and word correspondence features that we defined post-challenge), we achieved an *F*-score of 0.760. Including all our features further improved the *F*-score to 0.801. To assess the performance contribution of the different features sets, we retrained the system after removing each feature set in turn. Removal of the prior knowledge features or the statistical features resulted in a similar drop of performance (*F*-scores of 0.728 and 0.726, respectively). Leaving out the linguistic features reduced performance to some lesser extent (*F*-score 0.765).

Table 5 shows the performance results of the SVM classifier, using tmChem and Peregrine for entity normalization, on the CDR test set. For the CDR challenge, we submitted three runs using the SVM trained on the challenge features, in combination with tmChem and the Peregrine challenge system: one run used the decision threshold of 0.30 that resulted from our cross-validation experiments, the other two runs used thresholds of 0.20 and 0.40. The best *F*-score was 0.569, which was achieved for a threshold of 0.2. This result is higher than the *F*-score of 0.526 reported in the CDR challenge proceedings (13, 24). The reason is that the server showed occasional race-conditions during the challenge, which we only discovered and fixed after the challenge. Our system, which we named RELigator, ranked second among the systems of 18 participating teams in the CDR task (the best team achieved an F-score of 0.570) (13). Use of the improved, post-challenge Peregrine system only slightly improved performance (F-score 0.557 vs. 0.563 at a threshold of 0.3). However, the system trained with the additional post-challenge features yielded a considerably improved F-score of 0.602. For comparison, we also evaluated this SVM using the gold-standard entity annotations. This resulted in an F-score of 0.702.

**Table 5:** Performance of relation extraction systems on the CDR test data, for different entity annotations

System	Entity annotation	Threshold*	Recall	Precision	F-score
SVM, all challenge features	tmChem, Peregrine challenge	0.20	0.601	0.540	0.569
SVM, all challenge features	tmChem, Peregrine challenge	0.30	0.537	0.579	0.557
SVM, all challenge features	tmChem, Peregrine challenge	0.40	0.467	0.605	0.527
SVM, all challenge features	tmChem, Peregrine post-challenge	0.30	0.556	0.569	0.563
SVM, all post-challenge features	tmChem, Peregrine post-challenge	0.34	0.570	0.637	0.602
SVM, all post-challenge features	Gold standard	0.34	0.731	0.676	0.702

* Probability threshold for the SVM to decide whether there is a relationship.

## Discussion

We described our Peregrine-based system for disease normalization, and the RELigator system for CDR extraction. RELigator achieved an F-score of 0.526 for the CID challenge, which ranked second among 18 participating teams. Several post-challenge modifications of the systems resulted in a substantially improved F-score of 0.602 for CID, currently outperforming the best challenge submission. Evaluation of CID extraction using gold-standard entity annotations illustrates that the quality of entity recognition is still an important limitation.

Regarding the CDR extraction, our results indicate that knowledge-based features, statistical features and linguistic features each contribute to the final system performance, and thus contain at least partly complementary information.

Our original Peregrine system (F-score 0.757) was outperformed in the challenge by CRF-based disease recognition systems, with an F-score of 0.865 for the best performing system. The post-challenge modifications of Peregrine resulted in a substantial performance improvement (F-score 0.828). This result compares favorably with the F-score of 0.698 that we obtained in a previous study in which we also used Peregrine for disease concept recognition in a set of Medline abstracts (5). The lower performance in that study may partly be explained by the more demanding task to recognize disease concepts from any vocabulary in the UMLS, not just from MeSH like in this study.

Our error analysis revealed that most disease recognition errors were terminology-related. Inclusion of other vocabularies from the UMLS to increase the coverage of synonyms in combination with filtering on semantic types and manual term curation, may further improve Peregrine’s performance.

Remarkably, the gain in Peregrine performance before and after the challenge hardly increased the performance of the relation extraction pipeline (F-score rose from 0.557 to 0.563, using the challenge feature set and a 0.3 decision threshold of the SVM classifier). There may be several reasons for this. First, relation extraction performance is dependent on the performance of both the disease concept recognition and the chemical concept recognition. Improved disease recognition alone will therefore only be partially reflected in improved relation extraction. Second, disease recognition performance is based on the annotations of all unique disease mentions in the abstracts, whereas relation-extraction performance is based on disease annotations at the document level. The test contains 1988 gold-standard annotations of unique disease mentions and 865 gold-standard disease annotations that are part of CDRs. Again, improved performance of the disease recognition step is likely to be only partially reflected in improved relation extraction.

Despite the noisy entity data for instance generation, we still performed second in the challenge for CID extraction. Because the performance of relation extraction is not evaluated independently of entity recognition, it is hard to put the CID results into perspective. The task, in-part inspired by the needs of CTD curators, did not distinguish between DNER and CID performance, while this seems essential to bring this task forward.

The inter-annotator agreement (IAA) for the CID corpus is not known. Wiegers *et al.* (25) report a surrogate IAA score of 77% for annotation of chemical-gene interactions. This IAA averages agreement of each annotator against a gold standard, created by disagreement resolution, which presumably overestimates the true IAA. Our system has a micro-averaged F-score of 70% using gold-standard annotations, and may come within reach of the IAA. However, formal assessment of CID IAA needs to be performed.

Several improvements of the final model can be envisaged. The scope of syntactically connected chemical–disease pairs could be expanded through anaphora resolution. Governing and relating words could be encoded as word embeddings instead of nominal values, giving them a more compact and semantically rich representation. Simple token features in a window around chemical and disease could provide further context. Finally, the CDR annotations that we used to train our models were provided at the document level. We did not attempt to annotate the relation mentions in the document texts, which might have yielded stronger features.
